# The Role of Animal Models in the Study of Hematopoietic Stem Cell Transplantation and GvHD: A Historical Overview

**DOI:** 10.3389/fimmu.2016.00333

**Published:** 2016-08-30

**Authors:** Margherita Boieri, Pranali Shah, Ralf Dressel, Marit Inngjerdingen

**Affiliations:** ^1^Department of Molecular Medicine, Institute of Basic Medical Sciences, University of Oslo, Oslo, Norway; ^2^Department of Immunology, Oslo University Hospital, Oslo, Norway; ^3^Institute of Cellular and Molecular Immunology, University Medical Center Göttingen, Göttingen, Germany

**Keywords:** animal models, HSCT, aGvHD, cGvHD, pathophysiology

## Abstract

Bone marrow transplantation (BMT) is the only therapeutic option for many hematological malignancies, but its applicability is limited by life-threatening complications, such as graft-versus-host disease (GvHD). The last decades have seen great advances in the understanding of BMT and its related complications; in particular GvHD. Animal models are beneficial to study complex diseases, as they allow dissecting the contribution of single components in the development of the disease. Most of the current knowledge on the therapeutic mechanisms of BMT derives from studies in animal models. Parallel to BMT, the understanding of the pathophysiology of GvHD, as well as the development of new treatment regimens, has also been supported by studies in animal models. Pre-clinical experimentation is the basis for deep understanding and successful improvements of clinical applications. In this review, we retrace the history of BMT and GvHD by describing how the studies in animal models have paved the way to the many advances in the field. We also describe how animal models contributed to the understanding of GvHD pathophysiology and how they are fundamental for the discovery of new treatments.

The use of animal models to study human diseases is considered essential for understanding underlying pathophysiological and molecular mechanisms (1). Here, we will review how animal models have contributed to understanding the complexity of hematopoietic stem cell transplantation (HSCT) and graft-versus-host disease (GvHD). HSCT is the treatment of choice to cure many types of malignant and non-malignant hematological diseases. Despite continuous improvements in the pre- and post-transplantation procedures, the survival rate of transplanted patients is still poor. Acute GvHD (aGvHD) or chronic GvHD (cGvHD) represents major complications after HSCT with high mortality rates, in addition to other complications, such as relapse of the malignancy, engraftment failure, or opportunistic infections. GvHD is evoked by immunocompetent cells present in the graft that recognize and attack host tissue in an immunosuppressed environment.

## The History of Bone Marrow Transplantation

The advent of the atomic age in the early 1950s led to a strong interest in developing means to protect or cure the potentially lethal effects of radiation. Exposure to high doses of radiation was recognized to have deleterious effects on hematopoiesis and immune cell functions. By using different animal models including mice, rats, and guinea pigs, researchers soon discovered that injection of bone marrow or fetal spleen cells into lethally irradiated animals could reconstitute the hematopoietic system (1–5).

At the time, it was not clear how reconstitution occurred. At first, all evidence suggested the presence of “humoral factors” that stimulated regeneration of the endogenous hematopoietic system (6), but several studies in the following years showed that the newly formed hematopoietic system was in fact originating from the donor. In one study, biochemical techniques were used to track rat bone marrow cells transplanted into lethally irradiated mice. The authors postulated that the intravenously injected cells were able to migrate to the bone marrow where they survived and maintained their ability to proliferate and form a new hematopoietic system (7). In another study, Ford and colleagues used chromosomal markers to track the donor cells in the recipient. Their experiments provided the final evidence that reconstitution was originating from donor-derived cells (8). The responsible cells in the graft were identified almost 10 years later when Till and McCulloch in 1963 described a single progenitor cell type in the bone marrow with the potential to expand clonally and to give rise to all lineages of hematopoietic cells. This represented the first characterization of the hematopoietic stem cell (9).

In 1956, Barnes and Loutit proposed that an irradiation-transplantation approach could be used to treat fatal hematopoietic malignancies, such as leukemia (10). They speculated that irradiation followed by injection of bone marrow could treat leukemia if leukemic cells were as sensitive to radiation as normal cells. They also hypothesized, in the same paper, that if the entire population of leukemic cells was not eliminated by radiation, a cure could perhaps be achieved with the injection of cells capable to induce an immune response toward the residual leukemic cells. With this central paper, they introduced the concepts of therapeutic bone marrow transplantation (BMT), graft versus leukemia (GvL), and cell therapy.

At that time, it was already well known that grafts between individuals of different genetic backgrounds were rejected, while transplantations between inbred animals or identical twins were successful. The first successful human BMT was performed in 1959 by Thomas and co-workers who treated two leukemic patients with irradiation followed by infusion of bone marrow from their homozygous twins (“autologous” transplantation) (11). Despite successful transplantation, both patients experienced relapse. Further animal experiments and human transplantations demonstrated that irradiation followed by autologous BMT was not enough to eradicate leukemia. As an alternative approach, transplantation of immune cells derived from an individual or animal with a different genetic background was proposed (“allogeneic,” formerly termed “homologous”). This approach was experimentally tested in different mouse models (10, 12), resulting in successful eradication of the malignancies. Unfortunately, the mice died a few weeks later from what was then referred to as secondary or homologous disease. This disease was later defined as GvHD.

## Graft-Versus-Host Disease

The definition of GvHD is the result of a great number of accumulated observations since the 1940s. However, it was in particular the work of two independent researchers that elucidated the details of this phenomenon. Simonsen studied the acquisition of tolerance using chick embryos, and observed that injection of adult spleen or blood cells resulted in splenomegaly and severe hemolytic anemia in the recipient embryo. The rationale behind his experiments was that immunological competence is acquired after birth and, therefore, any immune effect in the adult to embryo transplantation setting is ascribable to the injected cells (13). During the same years, Billingham and Brent (14) performed similar studies in mice, describing splenomegaly, defects in growth, and early deaths when newborn mice were injected with allogeneic (“homologous”) adult lymphoid tissues. The phenomenon was termed the “runt disease” due to the growth retardation of the mice. In 1959, the same authors concluded that runt disease resulted from a graft-versus-host reaction (GVHR). Their observations were similar to those of experimental BMT (15–18). In addition, several other research groups at the same time described a reaction of grafted immune cells against the host (19–22), and by the beginning of the 1960s the GVHR was an established caveat for successful BMTs.

The nature of the GvHD reaction was ascribed to immunocompetent cells present in the bone marrow graft. Initial the first experiments showed that different hematopoietic cell populations could be fractionated by centrifugation on discontinuous albumin gradients (23). Factions with low content of lymphocytes and high content of blasts were shown to induce less GvHD (24). The lymphocytes responsible for inducing GvHD was identified as T cells, demonstrated by depletion experiments, first with the use of anti-lymphocyte serum (ALS) (25, 26), and later confirmed by the use of various methods to specifically remove T cells from the graft (27–30). These findings represented an important step forward in improving the success of BMTs. The removal of T cells from the bone marrow graft was soon applied in the clinic, and the depletion methods were substantially improved. Unfortunately, while a reduction in GvHD was achieved, patient survival was not improved, since the absence of T cells led to increased relapse, higher risk of infections, and diminished engraftment. To overcome the detrimental effects related to T cell depletions and to boost the GvL effect, donor T cells was re-introduced after BM transplantation. The infusion was delayed to allow establishment of tolerance toward the host. Murine and canine models served well in testing the timing and protocols for T cell infusions, now termed donor lymphocyte infusion (DLI) (31–34).

The success of allogeneic transplantation depends on the degree of histocompatibility match between donor and recipient. Research on outbred canine models has been vital to study genes involved in histocompatibility, and the importance of tissue typing and donor selection. In intial experiments, antisera were produced by cross immunization of dog littermates. These antisera were used in cytotoxicity tests in order to establish matched donor/recipient pairs that proved to be effective in reducing, but not eliminating, GvHD occurrence. The number and nature of the allo-determinants were still unknown; however, it was already clear that histocompatibility antigens were allocated to different loci and that the potential presence of different alleles would make the selection of donor–recipient pairs difficult, especially in unrelated animals (35–37). In the following years, several studies, especially on canine models, were vital for understanding the mechanisms related to histocompatibility. Histocompatibility was shown to be linked to a particular genomic region called the major histocompatibility complex (MHC).

Differences in the genes of the MHC region between donor and recipient are the major cause of T cell allo-activation and GvHD induction, but there are also other genes involved. The first observations of allo-antigens encoded outside the MHC complex came from the above-mentioned studies on BMT in dogs. In some instances, dogs developed GvHD when transplanted with MHC-matched bone marrow (37). Subsequent studies in the mouse provided more evidence on the involvement of non-MHC antigens (38–40), called minor histocompatibility antigens (41). GvHD induced through mHA was also T-cell mediated as demonstrated by a series of T-cell depletion experiments (42, 43), but the manifestation of the disease was delayed compared to classical GvHD.

Graft-versus-host disease can develop in two different forms that differ in pathogenesis, symptoms, and organ involvement. aGvHD affects up to 50% of the patients and accounts for 15% of post-transplantation mortality (44). Classically, acute GvHD (aGvHD) develops during the first 100 days after transplantation, but late acute aGvHD has also been described. Typical target tissues for aGvHD are the gastrointestinal tract, skin, and liver, but other atypical tissues include kidneys (45), salivary glands (46), oral epithelium (47, 48), and thymus (49). cGvHD develops later, and it occurs in ~50% of long-term survivors (50). Chronic GvHD (cGvHD) is associated with significant morbidity and mortality, and is still the leading cause of death in long-term survivors of HSCT (51). The organs involved are mainly skin, mouth, eye, and liver, and less frequently the gastrointestinal tract and lung. The pathogenesis of cGvHD is not clearly understood and the manifestations resemble more an autoimmune disease characterized by autoantibody production, chronic inflammation, and collagen deposition in target tissues.

## Acute GvHD

### Pathophysiology of Acute GvHD

**Figure 1:**
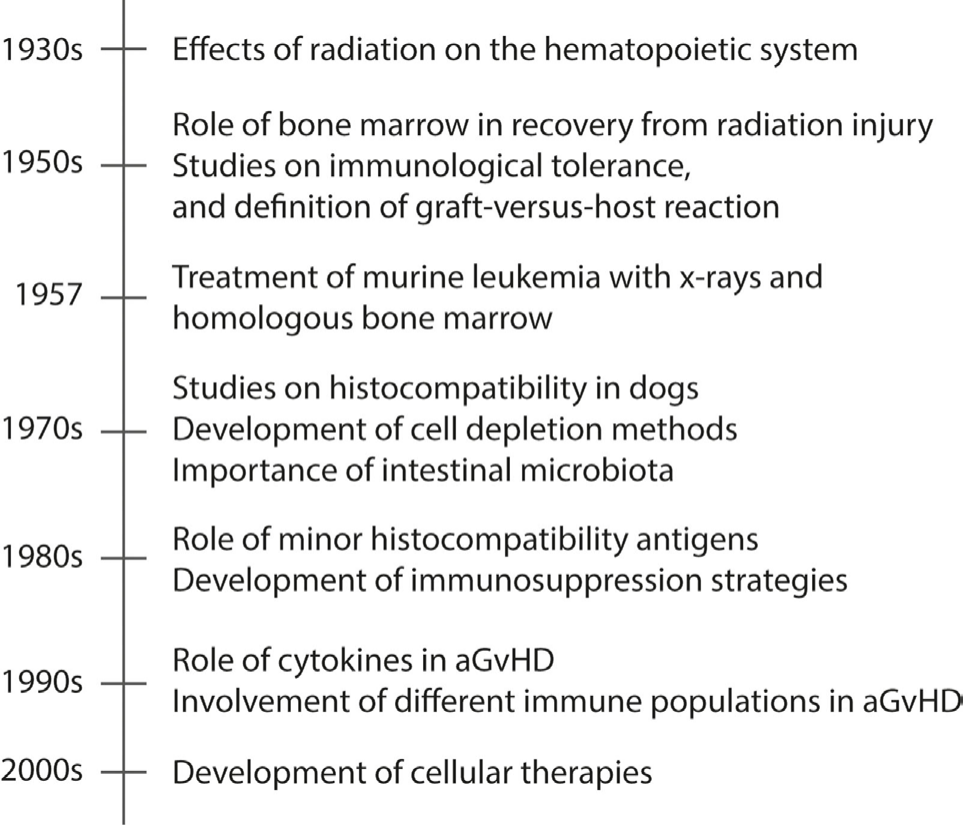
**A timeline presenting seminal events in animal models of aGvHD**. Acute GvHD is caused by activated alloreactive donor T cells that directly cause tissue damage in target organs, such as skin and gut. The timeline shows the seminal findings in animal models that have led to the current understanding of aGvHD pathology.

Understanding the complexity of the process leading to aGvHD requires in-depth mechanistic studies to identify the involvement of the different components of the immune system. For this reason, a great deal of the knowledge on the pathophysiology of aGvHD is derived from animal models. In this section, we will review the seminal findings from animal models that have led to the current view of how aGvHD develops, which is acknowledged to progress through three phases: (i) activation of antigen-presenting cells (APCs), (ii) allo-activation of donor T cells, and (iii) tissue destruction by alloreactive T cells. A summary of these findings are found in Figure 1, while an overview of rodent aGvHD models is found in Table 1.

**Table 1:** **Overview of rodent models for acute GvHD**.

Species	Model	MHC haplotype	Conditioning	MHC mismatch	Reference
Mouse	C57BL/6 → BALB/c	H2^b^ → H2^d^	TBI	Complete	(27, 30, 52, 53)
	C3H/HeJ → C57BL/6	H2^k^ → H2^b^			
	C57BL/6 → B10.BR	H2^b^ → H2^k^			
	C57BL/6 → B6C3F1	H2^b^ → H2^k/b^	TBI	Haploidentical	(54–58)
	C57BL/6 → B6D2F1	H2^b^ → H2^b/d^			
	C57BL/6 → B6AF1	H2^b^ → H2^b/a^			
	C57BL/6 → B6.C-H2^bm1^	H2^b^ → H2^bm1^	TBI or none	MHC-I	(59, 60)
	C57BL/6 → B6.C-H2^bm12^	H2^b^ → H2^bm12^		MHC-II	(59, 60)
	B10.D2 → DBA/2	H2^d^ → H2^d^	TBI	miHA	(42, 43, 61–63)
	B10.D2 → BALB/c	H2^d^ → H2^d^			
	B10 → BALB.b	H2^b^ → H2^b^			
	C57BL/6→ BALB.b	H2^b^ → H2^b^			
	DBA/2 → B10.D2	H2^d^ → H2^d^			
Rat	BN → LEW	RT1^n^ → RT1^l^	TBI or CYP or anti-CD25/CD154/CTLA4 Ig	Complete	(45, 64–71)
	PVG → BN	RT1^c^ → RT1^n^			
	DA → LEW	RT1^av1^ → RT1^l^			
	LEW → BN	RT1^l^ → RT1^n^			
	Wistar Furth → LEW	RT^1u^ → RT1^l^			
	LEW.1AR1 → LEW.1AR2	RT1A^a^, RT1B/D^u^, RT1C/E^u^ → RT1A^a^, RT1B/D^a^, RT1C/E^u^	TBI	MHC-II	(72)
	BN → (BN × LEW) F_1_	RT1^n^ → RT1^n/l^	TBI or none	Haploidentical	(47, 73)
	LEW → (LEW × BN) F_1_	RT1^l^ → RT1^n/l^			(48, 74–81)
	LEW → (LEW × DA) F_1_	RT1^l^ → RT1^l/av1^			
	PVG → (PVG × DA) F_1_	RT1^c^ → RT1^c/av1^			

#### The Conditioning Regimens Lead to Activation of APCs

In the first phase, both the conditioning regimen and the underlying disease play central roles. Together, they create the tissue damage responsible for the production and the release of pro-inflammatory cytokines and chemokines that activate macrophages and APCs. Of particular importance is the damage to the intestinal epithelium caused by the conditioning regimens, and the subsequent release of microbial products, such as lipopolysaccharide (LPS) by the resident gut bacteria (82–84). After HSCT, we face the uncommon situation in which APCs from both host and donors are present. Using mouse recipients whose APCs were unable to cross-present class I restricted peptides, Shlomchik and colleagues demonstrated how host, rather than donor APC, are presenting allo-antigens to donor T cells (85). APCs are activated by many signals released during this early phase of inflammation, where cytokines, such as TNF-α, IL-1, and IL-6, are central. These cytokines, apart from activating APCs, can also promote antigen presentation by non-professional APCs in the tissue and cause direct tissue inflammation that allows T cells to access their target tissues (86).

Total body irradiation (TBI) was the standard immunoablative procedure in the first years of BMT, and it is a widely used pre-conditioning method in animal models of HSCT. TBI is a very harsh procedure and causes significant damage to the fast-replicating tissues, such as skin and intestinal mucosa. Through research in murine and canine models, it was shown that the conditioning intensity and GvHD severity were directly correlated (83, 87, 88). Therefore, the development of milder conditioning regimens with less damage of the gut were rapidly developed in animal models and then brought to the clinic. At first, canine models showed that reduced intensity conditioning (RIC) led to graft rejection, but introduction of immunosuppression protocols post transplantation led to successful engraftment and reduced GvHD (87).

The role of the gut microbiota in the development of GvHD was first described in the early 1970s when experiments using germ-free mice showed that elimination of the gut microbiota reduced symptoms and mortality related to aGvHD (89, 90). In the following years, gut decontamination using broad-spectrum antibiotics was applied in clinical BMT. Results from clinical trials gave contrasting results and ultimately showed no increase in survival (91, 92). The reason is that the use of broad-spectrum antibiotics for gut decontamination does not take into consideration that the mutualistic relationship between patient and microbiota can be also protective in some instances. In more recent years, studies focusing on the composition of the microbiota have shown how the abundance of some bacterial species over others can protect or promote aGvHD. In particular, immunosuppressive treatments and aGvHD lead to loss in microbiota diversity, and the prevalence of members from the *Enterobacteriales* and *Enterococcus* order together with a loss in *Clostridiales* bacteria can promote aGvHD (93). The loss of *Clostridiae* species has important functional consequences since this population is thought to be an important promoter of regulatory T cell (Treg) proliferation and activity (94). The re-establishment of gut microbiota diversity through the introduction of probiotic therapy has been successful in reducing experimental aGvHD in mice (95).

#### Alloreactive T Cells Are Activated in Secondary Lymphoid Tissues

During the second phase, host APCs cross-present host auto-antigens to donor T cells, which will be activated and start proliferating. The interaction between APCs and T cells is further enhanced by cytokines produced in the first phase (96). Furthermore, co-stimulatory molecules, including CD80 and CD86 expressed by APCs and CD28 expressed on T cells, give the classical second signal required for full T cell activation. Their expression is upregulated by the ongoing inflammation. The secondary lymphoid tissues of the gut are thought to be the primary site of T cell activation, as shown by experiments demonstrating failure to develop aGVHD in mice lacking Peyer’s patches (PP) or where donor T cells lack the ability to migrate into PP (97).

The complex heterogeneity of T cell populations in humans makes it difficult to study the specific role of each subset, and how they may either promote or suppress aGvHD. Animal models are and have been essential for in-depth studies of the function of different T cell populations. For example, involvement of naïve rather than memory T cells in aGvHD has been investigated in mouse models. Several studies have shown how the transfer of purified effector memory CD44^+^CD62L^−^ T cells did not induce GvHD while retaining a GvL effect (98, 99).

CD4^+^ T helper (Th) cells can differentiate into diverse subsets depending on the cytokines and microenvironment they are exposed to, and different Th subsets may be involved in aGvHD pathogenesis in distinct organs (100): Th1 cells, producing IFN-γ, IL-2, and TNF-α, are mostly involved in the pathogenesis of gastrointestinal GvHD (101), while Th17 cells, producing IL-17A, IL-17F, IL-21, and IL-22, are thought to be the major pathogenic subset in skin GvHD (102). Only the simultaneous depletion of both these T helper populations is effective in controlling GvHD in mouse models (103).

The role of B cells in aGvHD is still controversial and under investigation. Host B cells have been shown, in mouse models, to be induced by TBI to produce IL-10 and contribute to reduce aGvHD occurrence (104). In previous studies in the rat, Renkonen and colleagues showed that, in lymphoid organs, there is increased B cell activation, proliferation, and antibody production early after BMT before the appearance of aGvHD symptoms. At later stages, the number of B cells decreased in the lymphoid compartment, but remained at high levels in the liver, suggesting a pathogenic role at least in this organ (105).

#### Alloreactive T Cells Migrate to Target Organs and Mediate Tissue Destruction

The third and last phase of aGvHD pathophysiology is the effector phase with migration of lymphocytes to their target tissues as one of the key steps. Chemokines and chemokine receptors specifically guide T cells in this process [reviewed in Ref. (106)]. CCR5 seems to have a broad effect as it has been described to mediate the recruitment of effector T cells, as well as Tregs, to many different target organs (97, 107, 108). In gastrointestinal aGvHD CXCR3 (109), CX_3_CL1 (110), and CCR6 (111) has been shown to play additional important roles. Blocking the interaction between chemokine receptors and their ligands is one of the therapeutic strategies that are currently under investigation. Once T cells reach their target site, the tissue destruction occurs by direct induction of apoptosis mediated by TNF-α and IL-1 (112), and/or by killing mediated by cytotoxic CD8^+^ T cells through perforin/granzyme and Fas–FasL interactions. The suppression of CD8^+^ T cell function is crucial in the control of aGvHD (113).

The effector mechanisms in aGvHD have been studied in several mouse models. Donor spleen cells lacking both perforin and FasL failed at inducing aGvHD (114). Using different genetic combinations of donor and host mice, Graubert and colleagues showed that the perforin/granzyme pathway is mostly involved in MHC class I restricted aGvHD, while the Fas–FasL interaction is involved in MHC class II restricted aGvHD (115). A more recent study showed that CD8^+^ T cells deficient for both perforin and FasL can still induce aGvHD in a donor–recipient combination that differs at a single MHC class I antigen. In this model the serum levels of IFN-γ and TNF-α were increased, and CD8^+^ T cells showed increased activation and proliferation. The authors concluded that both perforin and FasL are important during the contraction phase, and can contain the expansion of CD8^+^ T cells (116). T cell expressed TNF-related apoptosis-inducing ligand (TRAIL) induces pro-apoptotic signals upon binding to the TRAIL receptor on target cells, and is a commonly used killing pathway. Interestingly, this pathway has not been involved in tissue destruction in aGvHD, but it mediates anti-tumor responses. Murine T cells overexpressing TRAIL have been shown to suppress GvHD by inducing apoptosis of alloreactive T cells and mediating anti-lymphoma responses. The mechanism of action is thought to be through the interaction of the TRAIL^+^ T cells with host APCs bearing the TRAIL receptor DR5, but also fratricide of alloreactive T cells (117).

### Treatment of aGvHD

The current standard treatment for aGvHD is the use of steroids in combination with calcineurin inhibitors. This treatment induces general immunosuppression, but has side effects. In addition, many patients with aGvHD are resistant to this treatment. There is, therefore, a need to improve treatments and to target specifically aGvHD, without affecting GvL. The complex pathogenic mechanisms described in the previous sections offers a variety of pathways as potential targets for new therapeutic protocols. Also in the development of treatments for aGvHD, animal models have been and will be extremely important, although the translation from the pre-clinical to clinical setting is not always straight forward as human pathology is more complex due to many varying environmental factors as we will discuss in more detail below. Nevertheless, the possibility of studying the mechanisms involved in the efficacy of different treatments in animal models is instrumental for designing safe and effective protocols in humans. We will review some treatment strategies, where the use of animal models has been essential.

#### Immunosuppression

Canine models have been essential for testing post-transplantation immunosuppressive therapies to ameliorate GvHD. Together with immunohistocompatibility matching and T cell depletion, the use of immunosuppressive drugs in the post-transplantation phase represents one of the major advances for GvHD-free BMT. This is especially the case for partial MHC-mismatched transplantation or for non-myeloablative RIC regimens prior to transplantation. Methotrexate (MTX) is an immunosuppressive drug that targets the production of folic acid, which is essential for the synthesis of nucleic acids and proteins. MTX was first tested in dogs and proved to be effective at reducing GvHD occurrence (118, 119). A few years later, the discovery of calcineurin inhibitors (e.g., cyclosporine A and tacrolimus) greatly improved the prophylaxis protocols. When used early after transplantation, these drugs, alone or in combination with MTX successfully reduced GvHD occurrence in animal models (64, 74, 75, 120–122), and were soon after introduced to the clinic, where the combined use of MTX and cyclosporine showed an advantage over cyclosporine alone (123, 124). Although protocols vary between clinical centers, a combination of calcineurin inhibitors, MTX, and antithymocyte globuline (125) is still the gold standard (126).

#### Targeting of Cytokines

TNF-α is one of the most important cytokines involved in the pathogenesis of aGvHD, implicated in many steps during the disease progression. The importance of this cytokine in aGvHD was first described in a mouse model (127). Since then several studies have shown how neutralization of TNF-α can lead to reduced symptoms of aGvHD, and different means to target TNF-α and its receptor interaction either post- (128) or pre-transplantation are currently being explored.

IL-6 has a broad effect, activating many different immune cells. It has been associated with various inflammatory diseases, and has a predominant role in the early phases of aGvHD. Mouse studies have shown that IL-6 and its receptor (IL-6R) are upregulated during aGvHD (129), and that the addition of exogenous IL-6 can exacerbate the disease (130). Blockade of IL-6R were shown to reduce GvHD without affecting GvL (129, 131). Interestingly, mice treated with an anti-IL6R antibody also showed increased Treg reconstitution, which can effectively contribute to the reduction of aGvHD (131). A recently FDA-approved monoclonal anti-IL-6R antibody (Tocilizumab) has been shown to have beneficial effects in the treatment of steroid-refractory aGvHD (132).

The classical role attributed to IL-2 is to stimulate T cell proliferation. After many years of both experimental and clinical research, it is now clear that IL-2 has a more broad effect, including the maintenance of Treg homeostasis. Due to its effect on this regulatory population, IL-2 has been investigated as a therapeutic agent for treatment or prophylaxis of aGvHD. Administration of low doses of IL-2 alone has produced contrasting results in mouse models showing either beneficial (133) or no effects (134), but co-administration of rapamycin has been beneficial. Rapamycin targets conventional T cell signaling by blocking mTOR signaling. As Tregs use different signaling pathways, they are insensitive to rapamycin. In the presence of rapamycin, Treg do not compete with conventional T cells for IL-2 and this leads to their expansion (135).

The role of IL-18 and IL-22 in aGvHD pathogenesis is more controversial. ILC3 is a subset of innate lymphoid cells involved in maintaining gut homeostasis by producing IL-22, and is suggested to play a role in aGvHD pathogenesis. IL-22 is a cytokine with both protective and inflammatory functions, most likely depending on the microenvironment and the cell types involved (136). IL-22 produced by ILC3 targets epithelial cells, and regulates the production of anti-microbial factors, which are important in controlling the epithelial barrier function (137). In a mouse model, IL-22 depletion or deficiency in the host was shown to increase aGvHD severity, and ILC3 and IL-22 were suggested to protect the intestinal stem cell pool and epithelial barrier function during inflammation (138). Interestingly, in another mouse model, donor-derived IL-22 was shown to have the opposite effect and contribute to the severity of GvHD by promoting Th1 cell infiltration in presence of IFN-α (139).

#### Targeting of Chemokines and Chemokine Receptors

Blocking chemokine–chemokine receptor interaction is another logical therapeutic strategy that has been tested using animal models. Administration of anti-CXCR3 (140) or anti-CX_3_CL1 (110) antibodies in mouse models of aGvHD were shown to reduce gastrointestinal aGvHD. CCR5 is involved in migration of lymphocytes to several target tissue of aGvHD, and for this reason, it appears as an interesting target molecule. However, targeting CCR5 has given contrasting results as this chemokine is thought to be involved also in Treg recruitment to peripheral tissues (141).

Another interesting approach to treat aGvHD has been to take advantage of the upregulation of CXCL10 (ligand for CXCR3) observed in target tissues during disease. By injecting CXCR3-transfected Tregs, Hasegawa and colleagues showed specific migration of these cells to the target organs and subsequent reduction in aGvHD severity (142). Despite the encouraging results in animal models, it is important to keep in mind that the chemokine system is redundant, and blocking a single interaction does not always directly translate to a milder GvHD phenotype. For this reason, the use of agents with a broader effect that target more than one pathway has been tested. Among these, the broad-spectrum chemokine inhibitor NR58-3.14.3 has been successfully proved to reduce murine aGvHD especially in lung and liver (143).

#### Targeting of Co-stimulatory Molecules

Engagement of co-stimulatory molecules is necessary for full activation of T cells, and blocking these molecules has interesting potentials. Studies in animal models showed that anti-CD80 and anti-CD86 inhibited T cell expansion, and that mice treated with these antibodies experienced milder symptoms of aGvHD. Moreover, T cells isolated from CD28-deficient mice caused less severe GvHD (144, 145). Other studies have focused on targeting the CD40–CD40L pathway. Also in this case, the use of anti-CD40L antibodies reduced the severity of GvHD, which is thought to induce a selective depletion of activated T cells, and at the same time to induce Tregs (146–148). Along the same lines, the OX40–OX40L interactions are important in the pathogenesis of GvHD. T cells from rats with aGvHD upregulate OX40 (149), and administration of blocking antibodies against OX40L reduced aGvHD mortality in a mouse model (150). Other co-stimulatory pathways have been investigated in animals. Blockade of all of the following pathways have shown potential beneficial effects on aGvHD severity: 4-1BB/4-1BBL (151), ICOS/ICOS-L (152), LIGHT/HVEM (153), NKG2D-NKG2D-L (154), DNAM-1/DNAM-1-L (155), and the CD30/CD30L (156) pathways. However, co-stimulatory molecules are also important for the GvL effect, and blocking these molecules may severely compromise the GvL effect and, therefore, their clinical use may be limited. Nevertheless, experimental models suggest that not all molecules are equally involved in GvHD and GvL, and a better understanding of the importance of different co-stimulatory molecules for either GvHD or GvL may help identify new targets that can reduce GvHD while maintaining GvL.

#### Cell Therapy

Over the last 15 years, focus has been put on the use of cells with immunosuppressive functions to regulate aGvHD, in particular mesenchymal stem cells (MSCs) and Tregs (157). MSCs are found at very low frequencies in the BM and other organs, such as adipose tissue, placenta, and amniotic fluid, and have the potential to differentiate into adipocytes, chondrocytes, myocytes, and osteoblasts. MSC support hematopoiesis in the BM (158), and contribute to embryo implantation by promoting trophoblast invasion in the placenta (159). MSC also have immunosuppressive functions which, together with the ease at expanding them *ex vivo*, have made them promising candidates for immunotherapy for aGvHD [reviewed in Ref. (160)]. Despite the initial success in the treatment of steroid-refractory aGvHD (157, 161), MSC therapy has failed to give consistent results and animal studies also show contrasting outcomes (65, 66, 76, 162–167). Clinical trials testing the efficacy of MSC in the treatment of GvHD started before thorough investigation in pre-clinical models was completed (168). The precise mode of action of MSC on the immune system is not well understood, and these cells seem to acquire different functions according to the environment they are exposed to (169, 170). A better understanding of the biology of MSC, together with improved and standardized techniques for their isolation, characterization, and expansion may allow development of improved methods for their use in aGvHD prophylaxis or treatment. Tregs are a subset of CD4^+^ T cells that represent 5–10% of the total T cell pool in human and rodents (171–173). Tregs express high levels of CD25 and the transcription factor FOXP3, which is necessary and sufficient for their immunosuppressive activity (174). The functional and phenotypical properties of Tregs are conserved in human and rodent species, making animal studies particularly relevant for applications in humans. Treg have immunosuppressive properties, and they are fundamental to induce and maintain peripheral self-tolerance, protecting from aberrant immune responses that can lead to excessive inflammation and autoimmunity. Earlier animal experiments showed that depletion of Treg from the BM graft resulted in severe aGvHD, with mice dying by day 21 after transplantation compared to day 41 in non-depleted transplantations (175). Moreover, addition of donor Treg to the graft at 1:1 ratio with conventional T cells was shown to delay or prevent aGvHD (175, 176). In order to exert their effects, Tregs must migrate to the secondary lymphoid tissues where alloreactive T cells are activated. For this reason, only the CD62L^+^ and not the CD62L^−^ population of Tregs have been shown to protect from lethal aGvHD (177, 178).

Regulatory T cells are categorized into two groups, both important for controlling peripheral tolerance: naturally occurring Treg (nTreg) that develop in the thymus and induced Treg (iTreg) that differentiate from conventional T cells in response to TGF-β and IL-2. The second group is therapeutically interesting because iTreg can be generated *in vitro* from conventional T cells (179), and they can be expanded to therapeutically sufficient amounts. Unfortunately, animal studies have shown that this approach does not lead to any protection from aGvHD. The main reason is that iTreg are unstable *in vivo*, and upon transfer they can lose the expression of FOXP3, together with their immunosuppressive activity (180, 181). One of the hypotheses to explain this instability is that the inflammatory environment of aGvHD can induce the conversion of iTreg back to conventional T cells. In favor of this hypothesis are studies showing how blocking inflammatory cytokines, in particular STAT3-dependent cytokines, can improve the iTreg stability (182, 183). The nTreg represent, therefore, a potentially more effective therapeutic tool, but their low frequency in the periphery requires optimization of *ex vivo* or *in vivo* expansion protocols.

Natural killer cells are another therapeutically interesting cell population in context of aGvHD and GvL. Earlier studies in rodent models demonstrated that NK cells are important for successful engraftment after BMT. NK cells are particularly radioresistant and can mediate rejection of allogeneic cells (184–186). The presence of residual NK cells after immune ablation can play a role in the acceptance or rejection of the allogenic graft. Studies in rats showed that differences in both the classical and non-classical MHC class I genes can contribute to NK-cell mediated rejection (187, 188). On the other hand, the infusion of alloreactive NK cells, together with a reduced TBI in a haploidentical transplantation mouse model, caused eradication of leukemia and depletion of the residual host hematopoietic system, thus facilitating the engraftment of donor BM cells. The additional NK-cell mediated killing of host APC prevents activation of alloreactive T cells and, therefore, no aGvHD (189). The use of NK cells to cause a GvL effect is restricted to those combinations of donor–recipient in which NK cell alloreactivity can be fully exploited (KIR–MHC mismatch). Moreover, not all types of tumor cells have the same sensitivity to NK cells due to variable expression of ligands for activating and inhibitory receptors. Additional stimulation of NK cells with cytokines might be required in order to accomplish an effective and long-lasting GvL effect for the NK-cell resistant tumors. A recent study demonstrated how NK cells pre-activated with a combination of IL-12, IL-15, and IL-18 reduced aGvHD while retaining the GvL effect in a fully mismatched BMT mouse model. Injected NK cells retained their activated phenotype and exerted their immunosuppressive activity by inhibiting alloreactive T cell proliferation (190).

## Chronic GvHD

### Pathophysiology of Chronic GvHD

Chronic GvHD in the clinic was initially defined as any symptoms of GvHD that occurred more than 100 days after transplantation, but it became increasingly clear that this definition was inadequate. Due to the heterogeneity of the clinical manifestations of cGvHD, cGvHD was only properly defined a decade ago with the NIH Consensus Project on cGvHD, and cGvHD is now classified as a disease distinct from aGvHD (191, 192). Both aGvHD and cGvHD arise as a complication of allo-HSCT transplantation, but with different pathology and underlying disease-driving mechanisms. Hallmarks of cGvHD in the clinical setting are systemic fibrosis, chronic inflammation, sclerodermatous manifestations, and autoantibody production. These features are similar to several autoimmune diseases; yet do not fully mimic any particular autoimmune disease, being an entity on its own. However, due to the pathological similarities between cGvHD and autoimmune diseases, there has been a close synergy between the two fields; the difference being that cGvHD is mediated by a foreign donor lymphoid graft.

**Figure 2:**
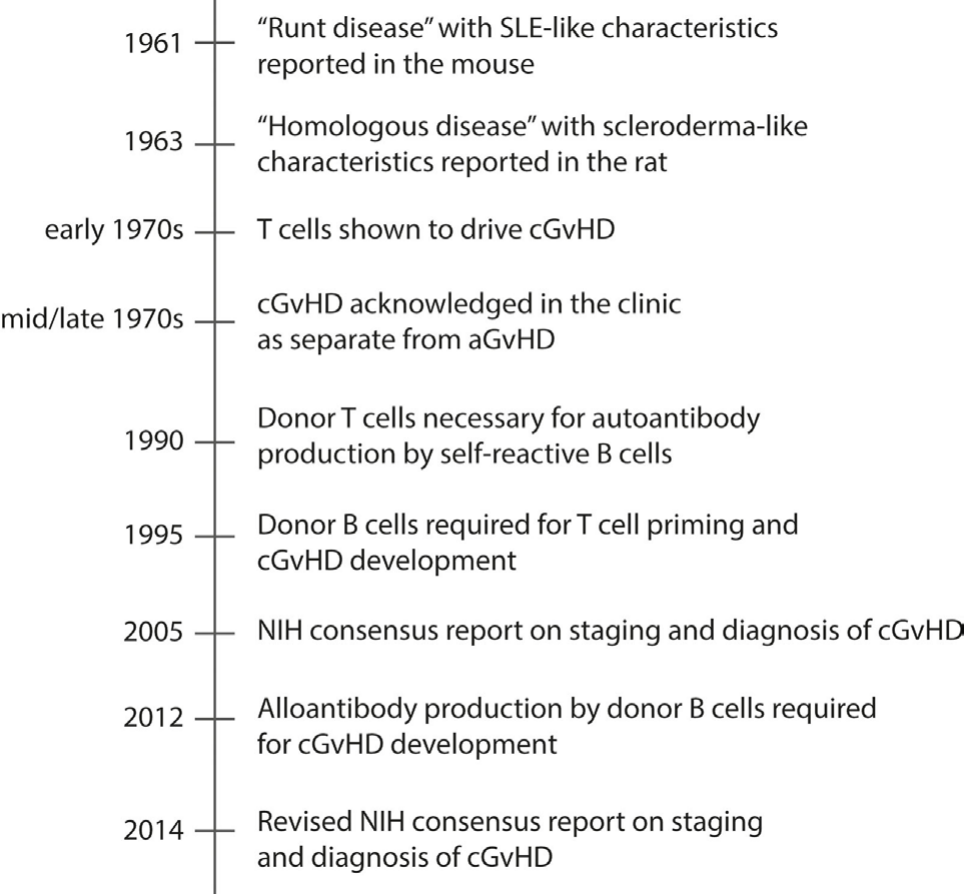
**Timeline of major events in cGvHD research using animal models**. Chronic GvHD is characterized by autoantibody production and deposition in target tissues, and tissue fibrosis. The timeline presents how animal models have contributed to increased understanding of the pathology of cGvHD, and included is also the NIH consensus reports on staging and diagnosis of cGvHD, which has contributed to development of improved rodent models of cGvHD during the last decade.

Therapies directed at ameliorating cGvHD have improved little over the past decades. The reason is incomplete knowledge of the underlying mechanisms that drive the disease. This has been mainly due to lack of animal models that completely recapitulate the full clinical heterogeneity of cGvHD. For more than three decades after cGvHD was acknowledged in the clinic, the best described and most utilized animal models for cGvHD addressed only one or a few of the many clinical manifestations of cGvHD, principally autoantibody generation or sclerodermatous disease (193) (Table 2). The clinical relevance of these animal models was a concern, as they did not fully mimic the clinical setting in terms of composition of the donor graft, preparative regimens, post-transplantation immune suppression, and the diverse human genetic background. Still, the animal models were instrumental for investigating subpopulations of donor and host leukocytes in the pathogenesis of cGVHD. In Figure 2, we have highlighted seminal findings from animal models. Moreover, during the last decade, improved models were developed that incorporate more of the features of clinical cGvHD, and these have led to further advances in our understanding of the underlying mechanism of cGvHD pathology.

**Table 2:** **Overview of rodent models for chronic GvHD**.

Species	Model	Conditioning	Manifestation	Reference
Mouse	C57BL/6 → B10.BR	Cy i.p./TBI	Bronchiolitis obliterans	(194)
	DBA/2 → BALB/c	TBI	Scleroderm.	(195)
	B10.D2 → DBA/2 × B10.D2 F1	TBI	Scleroderm.	(186)
	B10.D2 → BALB/c	TBI	Scleroderm.	(188, 189)
	C57BL/6 → BALB/c^a^	TBI	Scleroderm.	(190)
	BALB/c → BALB/c × A/Jax F1	None	SLE	(185)
	DBA/2 → DBA/2 × C57BL/6 F1	None	SLE	(183)
	CBA → CBA × A F1	None	SLE	(193)
	C57BL/6 → C57BL/6 × BALB/c F1	None	SLE	(193, 196)
	B6 → B6 × bm12 F1F1	None	SLE	(197)
Rat	LEW → SD	SD neonates tolerized with LEW lymphoid cells	Fibrosis	(184)

*^a^Low dose donor spleen cells prerequisite*.

#### Lupus-Like and Scleroderma-Like Animal Models of cGvHD

Chronic GvHD as a complication after allo-HSCT in the clinic was first acknowledged during the 1970s, with reports on autoimmune-like symptoms developing in patients several months after BMT. An autoimmune form of GvHD was described more than a decade earlier in experimental mouse and rat models. In 1961, Oliner, Schwartz, and Dameshek reported a form of GvHD (“runt disease”) in a parent to F1 hybrid transplantation model with autoimmune characteristics similar to systemic lupus erythematosus (SLE) (197). Two years later, Stastny, Stembridge, and Ziff reported in the rat a chronic form of GvHD (termed “homologous disease”) with features of sclerosing skin lesions similar to scleroderma (198). These works were followed by studies arguing that the “runting” syndrome of acute allogeneic disease must be separated from chronic allogeneic disease, the latter with symptoms manifesting at a later time point (199). It was suggested that, as for acute allogeneic disease, the chronic form was evoked by an immunological reaction of the donor against host antigens, although the exact mechanisms was not pinpointed at the time.

Throughout the 1970s and 1980s, the SLE-like and the scleroderma-like mouse models of cGvHD were the dominant animal models for cGvHD. These models were also extensively used for studies of autoimmune diseases. The SLE-like models generally involved transfers of lymphoid cells from a parental strain into non-irradiated F1 hybrids [e.g., BALB/c to BALB/c × A/Jax F1 (199) or DBA/2 to C57BL/6 × DBA/2 F1 (197)], resulting in transient or mixed chimerism. The main manifestation in these models is generation of autoantibodies, while skin pathology is less common. The relevance of these models has been questioned, mainly due to absence of bone marrow derived stem cells in the donor inoculum and absence of host immuno-depletion prior to transplantation.

Scleroderma-like mouse or rat models involves transplantation of major or minor MHC-matched or mismatched bone marrow into sub-lethally irradiated recipients, resulting in full donor chimerism (200–203). Here, the main manifestations are fibrotic changes in the skin, liver, lung, and salivary glands, while autoantibodies are less common. The scleroderma-like model for cGvHD shares many symptoms with sclerodermatous clinical cGvHD. The incidence of sclerodermatous cGvHD among long-term survivors of allo-HCST is around 3–10%, but the incidence of sclerodermatous cGvHD in the clinic is expected to rise as increasing numbers of unrelated donor transplants are performed, as well as the increased use of mobilized peripheral blood as stem cell source.

More recently, developed mouse models have better recapitulated human cGvHD. In these models, transplantation of MHC-mismatched T cell-depleted bone marrow together with a low dose donor lymphocytes leads to cGvHD (196). Fibrosis of the skin, salivary gland damage, and serum autoantibodies are observed. Similarly, a mouse model developed in Blazar’s laboratory with cyclophosphamide and lethal TBI pre-conditioning followed by allo-BM transplantation and low dose alloreactive T cell infusion, showed cGvHD manifestations in a wide range of cGvHD target organs (52). These models will likely significantly advance our understanding of the underlying immune reactions.

#### Donor-Derived CD4^**+**^ T Cells as Initiators of cGvHD

It was earlier shown that T cells play a central part for autoimmune development by collaborating with B cells for autoantibody generation (204). Fialkow and colleagues suggested that host CD4^+^ T cells were the drivers of autoantibody production, and that cGvHD was a purely host-derived, but graft-initiated, disease (205). However, it was soon clear that donor-derived CD4^+^ T cells were the real initiators of the disease, although the antigens recognized by the host-reactive donor T cells were not clear. Several pieces of evidence showed that naïve donor-derived CD4^+^ T cells were central for inducing cGvHD pathology, e.g., (i) when unfractionated lymph node cells or splenocytes were adoptively transferred into non-irradiated F1 hybrid hosts containing a mutated allele in MHC class I (B6 × bm1), a milder form of cGvHD was observed compared to transfer into F1 hybrid hosts with mutated MHC class II allele (B6 × bm12) (59), (ii) transfer of alloreactive donor CD4^+^ T cells obtained from mice with aGvHD to lethally irradiated secondary hosts led to cGvHD (206), and (iii) mature donor-derived CD4^+^ T cells were shown to cause both alloreactive and autoreactive responses using a DBA/2 to BALB/c cGVHD model (207). On the other hand, CD8^+^ T cells and the pool of CD4^+^ effector/memory T cells were found insufficient for inducing cGvHD (98, 208–211). Furthermore, depletion of CD8^+^ T cells from the graft, but not CD4^+^ T cells, led to autoantibody production. Later, a correlation was made between low CD8^+^ T cell numbers with cGvHD severity in several parents into F1 hybrid models (212). Thus, there is a notion that the frequencies of donor alloreactive CD8^+^ T cells may determine whether aGvHD or cGvHD develops. For example, CD8^+^ T cell anergy can shift the responses from an aGvHD to an SLE-like cGvHD (194, 213). Although CD8^+^ T cells are not necessary to induce cGvHD, they infiltrate skin and intestines where they contribute to the observed pathology (214).

#### B Cells as Autoantibody Producers and APCs

In contrast to aGvHD, B cells have a clear role in evoking cGvHD pathology. Although it was presumed that donor helper T cells were needed for production of autoantibodies by B cells, this was not directly shown until 1990, when Eisenberg’s group utilizing a mouse model of SLE demonstrated that autoantibody production by self-reactive host B cells, and not donor-derived B cells, was directly induced by donor-derived helper T cells (195, 215). The importance of B cells for inducing cGVHD pathology was subsequently shown by several investigators in SLE-like mouse models, by either blocking co-stimulatory molecules, such as CD40L and CTLA4, important for B-cell crosstalk (146, 216). Furthermore, B cell persistence, obtained by transferring perforin-deficient T cells from an aGvHD model (B6 into B6xDBA/2 F1 hybrids), resulted in a shift to cGVHD symptoms resembling SLE-like cGvHD (217). Later, in a mouse model of RIC, persistence of host B cells was associated with cGvHD lesions and autoantibodies of host origin (218). It was also shown that patients with extensive cGvHD had faster B cell recovery and detectable autoantibodies after allo-HSCT (219). Patients with severe cGvHD also have elevated levels of soluble B-cell activating factor (BAFF), which is evidence for activated B cells (220). Elevated BAFF serum levels were also associated with higher circulating levels of pre-germinal center (221) B cells and post-GC plasmablasts (222). Blockade of germinal centers with lymphotoxin-receptor Ig-fusion proteins was shown to suppress cGvHD, further demonstrating the involvement of mature, activated B cells (223). Interestingly, transplantation of bone marrow incapable of secreting allo-antibodies resulted in less severe cGvHD, demonstrating a role for both auto- and allo-antibodies in cGvHD pathology (223).

In addition to their role as producers of autoantibodies, B cells are potent APCs that stimulate donor T cells to further propagate the cycle that leads to cGvHD. Priming of donor T cells to mHA and subsequent cGvHD development was shown to depend on B cells as APCs (224). Almost a decade later, it was shown for the first time in a clinical setting, that a coordinated B and T cell response to a mHA, with donor B cells mediating the specificity, could be mounted in a setting of cGVHD (225). Further experiments in the mouse demonstrated that donor B cells promoted clonal expansion of autoreactive CD4^+^ T cells, their differentiation to the Th2 subset, and prolonged survival. In fact, these T cells mediate cGvHD when transferred into secondary recipients (226).

#### Mouse Models Suggest That cGvHD Is a Th2-Driven Disease

It has been debated whether cGvHD is primarily a Th1 or a Th2-driven disease. Most mouse models suggest that cGvHD is a Th2-driven disease. In the SLE-model, expansion of recipient B cells leading to lymphadenopathy, splenomegaly, and autoantibody production are observed. With this model, Th2 cytokines were shown to stimulate secretion of fibrosis-inducing cytokines (e.g., IL-13 and TGF-β) resulting in sclerodermatous disease (208, 209). When the cytokine balance was manipulated toward a Th1 type, a shift of symptoms to more aGvHD-like pathology was observed (227, 228). Furthermore, increased B cell activity was linked to increased levels of the Th2 cytokines IL-4 and IL-10, with concomitant suppression of IL-2 and IFN-γ by T cells isolated from animals with cGvHD (229). Confirming these observations, were clinical studies showing that a lack of Th1 responses led to early-onset cGvHD, and conversely, an early Th1 response with high IFN-γ production was associated with less cGvHD (230). These observations were later confirmed in mouse models, demonstrating lower incidence of cGvHD in the presence of donor T cells producing high levels of IFN-γ (221).

#### Involvement of Thymic Dysfunction for cGvHD Development

The thymus has a central role for both T cell development and for induction of T cell tolerance toward self antigens. Autoreactive T cells are negatively selected in this process. This is illustrated by studies of thymectomized neonatal mice that spontaneously develop multi-organ autoimmune disease (231, 232). Therefore, autoreactive T cells in context of cGvHD could result from defective tolerance induction due to thymic damage as a consequence of pre-conditioning or immune-mediated damage.

A mouse model of thymic dysfunction, where lethally irradiated hosts (C3H/HeN) receive T cell-depleted bone marrow from MHC-mismatched, MHC class II deficient donors (C57BL/6) represent a model where impaired negative selection occurs as a consequence of lack of MHC class II expression by thymic dendritic cells. In this model, many features of clinical cGvHD are observed, including sclerodermatous skin disease, weight loss, fibrosis, inflammation, and immune cell infiltration of salivary glands, while autoantibody generation is not reported (233). A weakness of this model is the fact that host thymic medullary epithelium cells also mediate negative selection. In addition, in this model thymic function is constitutively impaired by lack of MHC class II molecules, and does not address whether there is a temporal window of thymic damage where impaired negative selection occurs. In another model, where sub-lethal irradiation of BALB/c was performed prior to transfer of MHC-matched, mHA-mismatched DBA/2 bone marrow, donor T cells caused lesions characteristic of cGvHD when transferred to secondary allogeneic recipients. These cells were shown to be thymopoiesis dependent, and the authors, thus, concluded that T cells generated in the thymus were responsible for cGvHD development (206). Of note, a previous study using the same animal model could not demonstrate thymic dependence for cGvHD development (234).

A number of other mouse models points against a role for the thymus in the induction of cGvHD, as none of the murine models involving genetically unmodified mice has provided any evidence of impaired negative selection. In particular, no adversities of the thymic architecture or T cell development has been observed in the well-described SLE or Scleroderma-models described in the previous sections. Moreover, transfer of DBA/2 splenocytes and bone marrow to thymectomized BALB/c hosts did not change the incidence or the severity of cGvHD compared to mice with intact thymus (235). However, recent years’ research has indicated that alloreactive donor CD8^+^ T cells may damage thymic epithelial cells, leading to generation of autoreactive T cells (196, 236). The resulting autoreactive T cells were demonstrated to interact with donor B cells resulting in autoantibody production (196). Although recently developed mouse models strongly suggest that dysfunctional thymic negative selection is important for cGvHD pathogenesis, a role for the thymus in human cGvHD pathology is not clear. In addition, one must bear in mind that the thymus involutes by age, and older patients are not likely to have abundant functional thymic tissue.

### Treatment of cGvHD

Current treatment of cGVHD is largely based on immunosuppressive steroids, but development of more targeted therapies to replace or to treat steroid-refractory cGvHD are currently tested in pre-clinical animal models and several have now entered clinical trials.

#### Inhibition of Fibrosis

Platelet-derived growth factor (PDGF) and TGF-β are both pro-fibrotic cytokines inducing fibroblast activation. cGvHD patients are shown to have elevated levels of circulating, stimulating autoantibodies toward PDGFRα. PDGFR signaling leads to enhanced reactive oxygen species generation and subsequent collagen synthesis and deposition. Mouse cGvHD models were instrumental for developing Imatinib, a tyrosine kinase inhibitor that targets PDGFRα (41), and also anti-TGF-β treatment was shown to prevent skin and lung fibrosis (237). Imatinib has shown promising results in clinical trials of steroid-refractory cGvHD patients (238, 239). An enhanced effect was observed by simultaneous targeting of both PDGFRα- and TGF-β signaling pathways using Imatinib and Nilotinib, the latter targeting c-Abl in the intracellular pathway induced by TGF-β (240).

#### Targeting of B Cells

As donor-derived B cells are central auto- and allo-antibody producers, and significantly contribute to clonal expansion of donor-derived CD4^+^ T cells, therapies have been directed at depleting B cells from the patients. The well-known B cell-depleting antibody Rituximab (anti-CD20) specifically targets B cells and has been used in the treatment of patients with refractory cGvHD, resulting in objective improvements of symptoms (241–243). However, the antibody rarely results in complete remission of cGvHD. It is also a concern that anti-CD20 antibodies poorly target germinal centers in lymph nodes, in contrast to efficient removal of B cells from peripheral blood (244).

#### Infusion or Induction of Tregs

As for aGvHD, the use of Tregs in therapy of cGvHD is being exploited in clinical trials, as cGvHD patients have reduced frequencies of Tregs similar to aGvHD patients (245, 246). In mouse models, it was shown that transfer of *ex vivo* expanded Tregs resulted in suppression of cGvHD (247), suggesting that they may be utilized to treat cGvHD. However, the required *ex vivo* expansion of Tregs to obtain sufficient numbers for transfer into patients is technically challenging, and may also be associated with changes in their functionalities as discussed above. Another strategy is the expansion of Treg *in vivo* by injecting low-dose subcutaneous IL-2 leading to increased Treg accumulation that has demonstrated reduced severity of cGvHD (248).

## Advantages and Limitations of Animal Models for GvHD

As outlined above, animal models have largely contributed to current GvHD prophylaxis and treatment protocols (249). An overview of the most common animal models are found in Tables 1 and 2. Each model has advantages but also their limitations.

In the early days of GvHD research, canine models were important for studying the role of MHC disparities in GvHD (250), and the canine models substantially contributed to advance our understanding of the biological mechanisms at play in HSCT and GvHD. Among the many researchers in this field, Edward Donnal Thomas is often recognized as the father of clinical BMT, for which he earned the Nobel Prize of Medicine in 1990. In addition to his clinical work, he carried out intensive research in canine models of BMT and GvHD. Canine models are still used in studies pertaining to the effectiveness of cellular immunotherapy, such as the utility of an anti-CD28 antibody as therapy to prevent GvHD during allo-HSCT (251).

Although outbred animal models are sometimes required to better mimic several aspects of human HSCT and GvHD, the most preferred animal model in context of GvHD is currently the mouse (252). The advantages of mouse models are the (i) broad availability of transgenic and gene-deficient strains that provide mechanistic insights into the role of individual genes for GvHD (253), (ii) the presence of inbred strains that are well characterized for studying GvHD and GvL, (iii) the availability of many well-characterized reagents, and (iv) the relative low costs of breeding mice (254, 255).

Several well-characterized mouse models of both acute and cGvHD have been established, such as the full MHC class I mismatch C57BL/6 to BALB/c (256) or C3H/HeJ to C57BL/6 (30) for aGvHD, and B10.D2 to BALB/c for cGvHD. The mouse is a particular valuable model to determine the role of individual cell types, genes and factors that affect GvHD. Examples are transgenic mice that have a mutant MHC class I, e.g., B6.C-H2^bm1^ (bm1), or mutant MHC class II, e.g., B6.C-H2^bm12^ (bm12). Both the H2^bm1^ and H2^bm12^ models have been important in understanding the interaction of T cells with recipient and donor APCs (257). Humanized murine models are also interesting models for GvHD and GvL research (258). An example is the Hu-PBL-SCID model, which is based on the NOD-*scid* mice. In this model, HIV-1 envelope protein gp120 delayed GvHD development by activation of human Tregs (259). Similarly, GvHD development was delayed in the Hu-PBL-SCID model based on NOD-*scid IL2^null^* mice following treatment with a soluble Fas ligand (260). On examination of the kinetics of engraftment and development of GvHD in the latter model, it was observed that mice deficient in MHC class I exhibited a delay in GvHD (261). However, it is difficult to select an appropriate model, as engraftment or the strength GvHD symptoms does not necessarily correlate with the pathophysiology of GvHD in humans (262).

Rats are also used for GvHD studies. Rats are genetically similar to mice, but they are larger in size, have a longer life span, and have more biomaterial that can be used for experiments (263). GvHD models in rats include MHC-mismatched strains between LEW and BN (264, 265), or between PVG and BN (67, 68). Rat models have been used to test immunomodulatory drugs such as Thalidomide (266) and MC1288, an analog for vitamin D (267) as therapeutic strategies for GvHD.

Conditioning prior to transplantation causes tissue damage and pro-inflammatory responses that affect the GvHD outcome (268, 269). Therefore, the timing of transplantation and conditioning regimens will significantly affect the experimental outcome (270). Conditioning regimens in murine models frequently involves TBI, in contrast to the clinical settings where patients are usually given chemotherapy, and where only a few patients are subjected to TBI (271). Sadeghi and colleagues developed a chemotherapy-based GvHD mouse model with busulfan and cyclophosphamide as the conditioning regimen. The mouse model was mismatched for both MHC and mHA [C57BL/6 (H2^b^) to BALB/c (H2^d^)], and the allogeneic transplanted mice developed clinical and histological symptoms associated with GvHD, such as apoptosis and T cell infiltration into the target organs (272). This model represents a myeloablative-conditioning regimen, which is most commonly used in the clinic. Another mouse model involving the same chemotherapy as conditioning was described using MHC-matched, mHA-mismatched mice [LP/J (H2^b^) – C57BL/6 (H2^b^)]. This model was developed to more closely mimic the clinical situation, where patients usually are MHC matched. With this model, similar T cell infiltration, GvHD-specific damage, and systemic inflammation were observed in the mice as reported in humans (273). Thus, animal models of selective mHA mismatch may represent human HSCT more closely than MHC-mismatched models (193).

Another important consideration is the fact that the immune cell compositions vary between species. In murine models, mice receive bone marrow and T cells from an allogeneic counterpart to induce severe aGvHD. The T cell expansion is mainly homogeneous in the inbred recipients, in contrast to the heterogeneous T cell response in humans (274). Furthermore, differences in the proportion of lymphocyte subsets (such as CD4^+^, CD8^+^, and Tregs) between species can influence pathophysiology of GvHD (193). In addition, the metabolism and pharmacology of animal models can be different and these differences between animal models and humans could explain why some of the findings in mice models have not been successfully translated into clinical trials. For example, IL-11 reduced transplant related mortality (TRM) and prevented GvHD while maintaining GvL effects in mice (275). By contrast, IL-11 included as GvHD prophylaxis caused multi-organ failure in a phase I/II double blinded, placebo-controlled trial for allo-HSCT (276). In another example, experiments in mice showed that GvHD was effectively prevented in animals by therapy with a monoclonal antibody against the IL-2 receptor (IL-2R) (277). However, the use of IL-2R antibodies in two separate clinical trials was only moderately successful in reducing the incidence of severe GvHD (278, 279).

An important difference between mouse and rat animal models and humans is the homogenous genetic composition of inbred rodents, in contrast to the heterogeneous humans (193). Furthermore, the genetic drift that occurs in inbred strains from a particular colony might affect the ability to reproduce data consistently between labs (280). Given that inbred strains are an artificial model, several different inbred strains and/or outbred animals should be used to better represent the genetic complexity in the human population. For this reason, canines are sometimes preferred to study new regimens in prophylaxis and treatment of GvHD (281).

Moreover, there are important species differences that need to be taken into consideration when extrapolating results found in animal models to humans (282). Differences in the anatomy, physiology, microbiota, play an important role in GvHD pathology (193). In addition, age plays an important role in influencing the efficacy of immune reconstitution post-transplant, as well as susceptibility to GvHD (283). Non-human primates or canine models are better fit for long-term therapies, given their longer life span than rodents. Moreover, the effects of opportunistic infections that affect HSCT outcome that can be observed in humans are not modeled in rodents kept in SPF conditions.

An alternative to study GVHR is the use of the skin explant model. The skin explant model can closely mimic the *in vivo* mechanisms and pathology of human GvHD (284, 285). The skin explant assay for GvHD was initially tested as a method to predict incidence and severity of GvHD in humans (286), and we have previously shown that a rat skin explant assay for GvHD is useful to determine the severity of GvHD between different rat strains (287). Although *in vitro* studies can provide hypotheses and models for research, there is a strong need for testing and validation in an *in vivo* animal model. The important pathophysiological conditions and symptoms of GvHD have been successfully reproduced in a number of animal models (288), and animal models have been very useful in understanding various key mechanisms of GvHD and GvL. However, they still fail to fully compensate for the variable time of onset of the disease, the rate of progression, relapse of primary disease, and other important clinical variables attributed to GvHD pathology and HSC outcome (288). Till date, researchers have failed to create accurately an animal model encompassing all human parameters (289). Identifying suitable models for specific fields would be beneficial.

## Future Perspectives

Overall, substantial progress has been made using animal models to understand GvHD. However, major clinically relevant questions still remain unanswered. It is important to understand the mechanisms involved in the effect of RIC on late-onset aGvHD, for instance, or the mechanisms involved in steroid-resistant disease (290). In spite of distinctive similarities of GvHD pathology between different animal models and humans, the corollary question remains: Do animal models, in absence of immunosuppressive medications post transplantation, adequately simulate GvHD that occurs in humans (291). Designing interventions using animal models involving mimicry of the experience of the patient during their treatment in the clinic could be important. Larger animal models or non-primate humans could be used to investigate steroid resistance, secondary treatments, and also monitor these effects long term.

## Author Contributions

MB, PS, and MI wrote the manuscript; MB, PS, RD, and MI planned the contents; RD and MI reviewed the contents.

## Conflict of Interest Statement

The authors declare that the research was conducted in the absence of any commercial or financial relationships that could be construed as a potential conflict of interest.
